# High-Level Overproduction of *Thermobifida* Enzyme in *Streptomyces lividans* Using a Novel Expression Vector

**DOI:** 10.3390/ijms140918629

**Published:** 2013-09-10

**Authors:** Jun-Xia Li, Long-Mei Zhao, Ru-Juan Wu, Zhao-Jun Zheng, Ri-Jun Zhang

**Affiliations:** State Key Laboratory of Animal Nutrition, College of Animal Science and Technology, China Agricultural University, Beijing 100193, China; E-Mails: lijunxia_cau@163.com (J.-X.L.); zhaolongmei@126.com (L.-M.Z.); lingxiabenpao@aliyun.com (R.-J.W.); zhaojun064@163.com (Z.-J.Z.)

**Keywords:** *Streptomyces lividans*, *Pichia pastoris*, Endoglucanase, *Cel6A*

## Abstract

In this study, we constructed a novel *Streptomyces*-*E.coli* shuttle vector pZRJ362 combining the xylose isomerase promoter and amylase terminator. A gene encoding the endoglucanase Cel6A in *Thermobifida fusca* was amplified by PCR, cloned into *Streptomyces lividans* host strain using the novel expression vector and *Pichia pastoris* GS115 host strain using the vector pPICZα-C, respectively. Afterwards, the expression pattern and the maximum expression level were comparatively studied in both expression systems. The maximum enzyme activity of Cel6A-(His)_6_ secreted in *S. lividans* supernatant after 84-h of cultivation amounted to 5.56 U/mL, which was dramatically higher than that secreted in *P. pastoris* about 1.4 U/mL after 96-h of cultivation. The maximum expression level of Cel6A-(His)_6_ in *S. lividans* supernatant reached up to 173 mg/L after 84-h of cultivation. The endoglucanase activity staining SDS-PAGE showed that there were some minor proteins in *S. lividans* supernatant which may be the Cel6A derivant by proteolytic degradation, while there was no proteolytic product detected in supernatant of *P. pastoris*.

## 1. Introduction

Microbial exploitation for the production of industrial enzymes like keratinase, pectinase, cellulase, xylanase, and lipase has attracted more and more attention. Actinomycetes constitute a formidable group of industrially important microorganisms that have been explored for the production of a variety of enzymes. A large number of enzymes have been found and isolated from actinomycetes, including phospholipase [1], phosphatase [2], cellulase [3], keratinase [4], protease [5,6], α-amylase [7,8], and xylanase [9–11], and some genes of them were cloned and expressed in *Escherichia coli* [12–16], *Pichia pastoris* [7,12,17] and *Streptomyces lividans* [1,13,14,18]. Comparision of the productions of these proteins in the three hosts revealed that the expression of active proteins achieved in *P. pastoris* and *S. lividans* was much higher than that achieved in *E. coli*, even expression of some actinomycetes enzymes in *E. coli* was unsuccessful. With respect to comparision of the expression yield in *P. pastoris* and *S. lividans*, the results obtained to date showed the expression yield of *P. pastoris* would seem to be superior to those of *S. lividans* [19–21], despite relevant differences between *P. pastoris* and actinomycetes. Theoretically, owing to similarities in their codon usage, with C or G preferentially as the third base of most codons, *Streptomyces* could be an ideal host for the expression of genes from actinomycetes, gram-positive bacteria with a high content of G + C nucleotides [22]. The most possible explanation for the low yield in *Streptomyces* was the inefficient expression vector or promoter. Thus, it is highly desirable to develop an efficient expression vector in *Streptomyces* to overproduct proteins of interest from actinomycetes, especially overexpress the thermostable enzyme from the thermophilic genera, such as *Thermobifida* and *Thermoactinomyces* because of their overall inherent stability.

Among the thermophilic actinomycetes, *Thermobifida fusca* is a major degrader of plant cell walls owing to its abundant extracellular cellulases and xylanases [23]. In this study, one gene *cel6A* (GenBank accession No. M73321) encoding an endoglucanase was selected to study the capacity of the novel expression vector we constructed in this paper. The reason for selecting endoglucanase Cel6A lies in the potential industrial application because of its thermostability, utility through a broad pH range and high activity [24].

Although an expression vector for production of endoglucanase Cel6A from *T. fusca* YX in *Streptomyces* has been reported recently, its maximum expression level was relatively low (about 64 mg/L) [25]. Therefore, the aim of this study was to construct a *Streptomyces*-*E. coli* shuttle vector for overproduction of the endoglucanase Cel6A from *T. fusca* and to compare it with the production obtained in *P. pastoris*. In the course of these experiments, comparisons of the expression pattern, the maximum expression level in both expression systems were investigated.

## 2. Results and Discussion

### 2.1. Characteristics of pZRJ362

**Figure 1:**
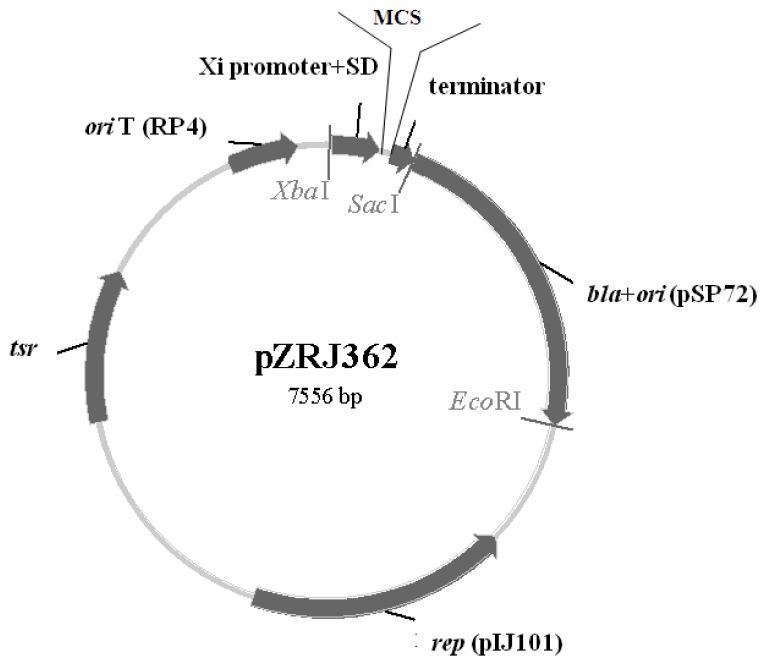
Map of the expression vector pZRJ362. *bla*, Ampicillin-resistance gene; *tsr*, Thiostrepton-resistance gene; MCS, Multi-cloning site; *ori*, Origin for *Escherichia coli; rep*, Replication gene in *Streptomyces; ori*T, Origin for transfer.

As presented in Figure 1, an object gene can be cloned into the MCS, located between the promoter and terminator of the pZRJ362 vector. Moreover, this vector can be transformed using intergeneric conjugation between *E. coli* and *Streptomyces* because of *ori*T. The vector was designed to enable selection with Amp and Thiostrepton antibiotic in *E. coli* and *Streptomyces*, respectively.

### 2.2. Cloning and Expression of *Cel6A* in *Streptomyces lividans*

**Figure 2:**
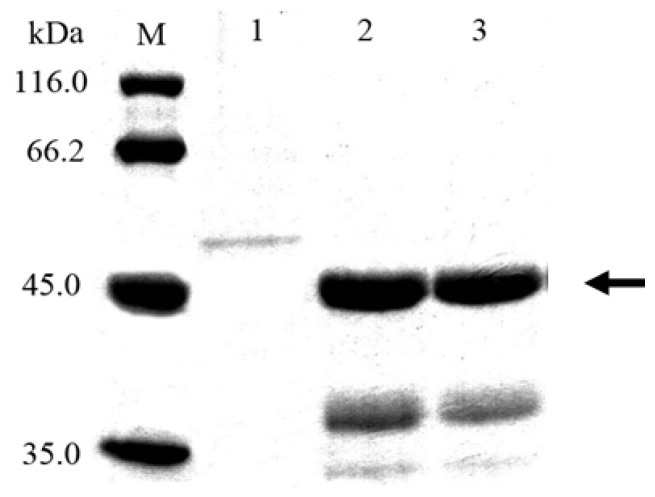
SDS-PAGE analysis of culture supernatants of *Streptomyces lividans* transformants. 1, Supernatant of transformant harboring pZRJ362; 2, Supernatant of transformant harboring pZRJ362-*cel6A*; 3, Supernatant of transformant harboring pZRJ362-*cel6A*-(His)_6_; M, Molecular weight marker. All samples containing 16 μL supernatant were loaded onto a 15% polyacrylamide gel.

The expression plasmids, pZRJ362-*cel6A* and pZRJ362-*cel6A*-(His)_6_, containing approximately 1700 bp gene sequence which encoded endoglucanase Cel6A transcribed under the control of the *Actinoplanes missouriensis* xylose isomerase promoter, were conjugated in *S. lividans* and the plasmid vector pZRJ362 was used as a negative control. The resultant *S. lividans* transformants were cultured in TSB medium for 96 h. As a result, a large amount of *T. fusca* Cel6A or Cel6A-(His)_6_ was secreted in *S. lividans* supernatants using its signal sequence, and the enzyme activity in supernatants amounted to 5.06 and 5.20 U/mL, respectively. SDS-PAGE staining with Coomassie Blue (Figure 2) confirmed the expression of Cel6A. As shown in Figure 2, a protein band with a molecular mass of about 45 kDa was detected in both supernatants of the *S. lividans*/pZRJ362-*cel6A* (molecular mass, 43 kDa) and *S. lividans*/pZRJ362-*cel6A*-(His)_6_ (calculated molecular mass, 43.8 kDa) transformants as the major protein but not in culture of *S. lividans* transformed with pZRJ362. Analysis by SDS-PAGE revealed that the target proteins were exceptionally highly expressed and secreted in *S. lividans* using its own signal sequence, constituting up to 80.6% of all soluble protein in supernatants (determined by Bandscan 5.0 software (Glyko, Novato, CA, USA)).

**Figure 3:**
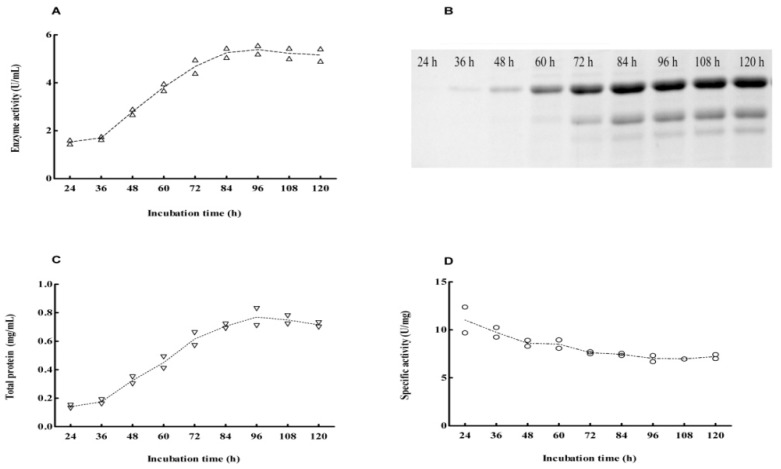
Time course of enzyme activity and expression of Cel6A during cultivation of *Streptomyces lividans*/pZRJ362-*cel6A*-(His)_6_. (**A**) Endoglucanase activity; (**B**) Expression of *Cel6A*-(His)_6_; (**C**) Total protein; (**D**) Specific activity. Endoglucanase activity (U/mL) was assayed using CMC-Na as substrate. The specific activity of supernatant was calculated as U/mg total protein. Total protein concentration was measured by Bio-Rad Protein Assay kit (Hercules, CA, USA).

Comparision of the specific activity of Cel6A (6.48 U/mg) and Cel6A-(His)_6_ (6.26 U/mg) in the supernatant after 96-h of cultivation showed the hexahistidine tag at the *C*-terminal has no adverse on the activity of Cel6A. Therefore, we merely selected *S. lividans*/pZRJ362-*cel6A*-(His)_6_ for purification and further study. Concurrent with the growth of *S. lividans*/pZRJ362-*cel6A*-(His)_6_ transformant, the measurement for endoglucanase activity and total protein content in the supernatants was taken. The maximum endoglucanase activity was observed at 84-h of cultivation (5.56 U/mL) and the production of Cel6A-(His)_6_ remained basically constant at longer incubation time (Figure 3A). The accumulation of 45-kDa Cel6A-(His)_6_ protein in the supernatant obtained at different culture time was also confirmed by SDS-PAGE analysis (Figure 3B). Unlike the time course of enzyme activity, the total protein content reached a maximum level after about 96-h (0.81 mg/mL) (Figure 3C) and it was slightly decreased by incubation, probably due to the degradation by a bit of endogenous protease of *S. lividans* 1326. Based on the result that the specific activity of supernatant cultured 84-h was slightly higher than that cultured 96-h (Figure 3D), we chose the supernatant at 84-h of cultivation for further purification.

**Figure 4:**
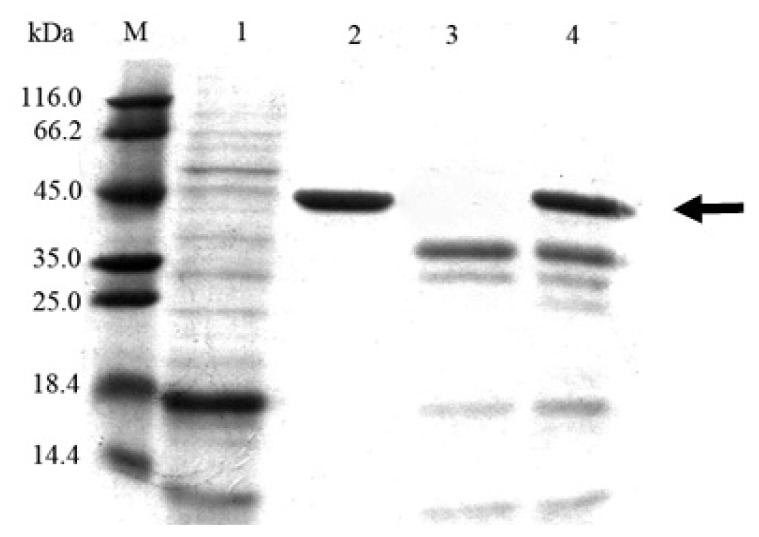
Purification of Cel6A-(His)_6_ from culture supernatant. 1, Supernatant of *Streptomyces lividans*/pZRJ362; 2, Purified Cel6A-(His)_6_; 3, The pass-through fraction from the (His)_6_-tag affinity column; 4, Supernatant of *S. lividans*/pZRJ362-*cel6A*-(His)_6_; M, molecular weight marker.

The SDS-PAGE of the purified enzyme exhibited a single band after (His)_6_-tag affinity column purification (Figure 4). Estimated from the specific activity of purified Cel6A-(His)_6_ (32.3 U/mg), the maximum expression level of Cel6A-(His)_6_ reached to 173 mg/L after 84-h of cultivation of *S. lividans* harboring pZRJ362-*cel6A*-(His)_6_. With the combination of xylose isomerase promoter and amylase terminator, the protein concentration of the secreted Cel6A-(His)_6_ was approximately 3-fold higher than that with the combination of *Streptoverticillium cinnamoneum* phospholipase D promoter and terminator [25].

### 2.3. Cloning and Expression of *Cel6A* in *Pichia pastoris*

**Figure 5:**
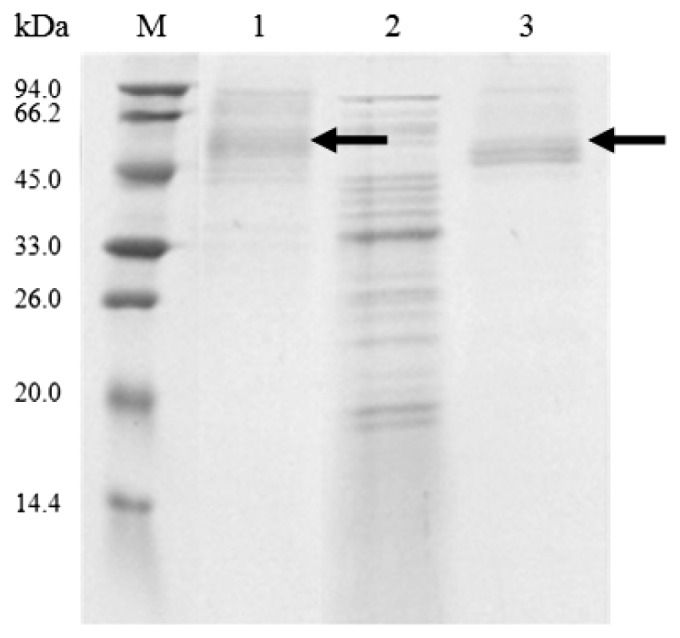
SDS-PAGE analysis of proteins produced by *Pichia pastoris* transformants. 1, Supernatant of transformant harboring pPICZα-*cel6A*-(His)_6_; 2, Supernatant of transformant harboring pPICZα-C; 3, Supernatant of transformant harboring pPICZα-*cel6A*; M, Molecular weight marker. All samples containing 16 μL supernatant were loaded onto a 15% polyacrylamide gel.

The plasmid pPICZα-*cel6A*-(His)_6_ or pPICZα-*cel6A* carrying the mature *cel6A* gene was regulated by a promoter of alcohol oxidase gene and the production of Cel6A was induced with 0.5% methanol. Using pPICZα-*cel6A* or pPICZα-*cel6A*-(His)_6_, the maximum Cel6A activity in the BMMY medium reached approximately 1.6 U/mL and 1.4 U/mL after 96-h of cultivation, respectively. Figure 5 showed the SDS-PAGE analysis of proteins secreted into the culture supernatants by *P. pastoris* transformants. Several protein bands corresponding to a molecular mass about 55 kDa were observed, probably because of the formation of different numbers of glycosylated side chains. The result is consistent with the previous studies which indicated the generation of several glycosylated proteins in the supernatants of *P. pastoris* transformants [19]. Analyses of SDS-PAGE showed that the target proteins Cel6A-(His)_6_ and Cel6A accounted for 59.6% and 54.7% of all soluble protein in the supernatants (determined by Bandscan 5.0 software), respectively. As seen in SDS-PAGE gels, the expression level of Cel6A in *P. pastoris* was quite low in comparison with that in *S. lividans*. Therefore, for *T. fusca* endoglucanase Cel6A, the use of *S. lividans*/pZRJ362 as an expression system represents a dramatic improvement in comparision with the use of *P. pastoris/*pPICZα-C.

### 2.4. Expression Pattern of *Cel6A* in *Streptomyces lividans versus Pichia pastoris*

**Figure 6:**
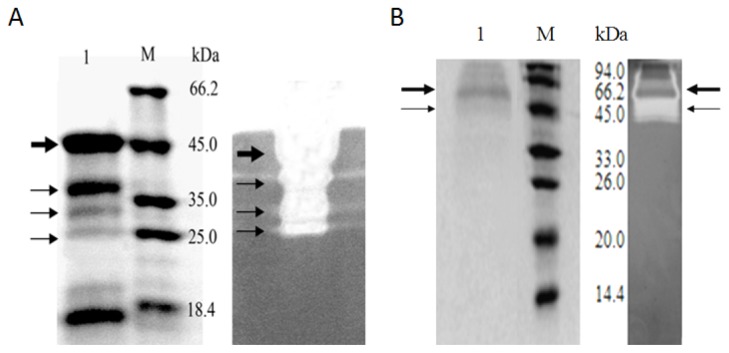
SDS-PAGE and activity staining analyses of expression pattern of Cel6A-(His)_6_ in *Streptomyces lividans* versus those in *Pichia pastoris*. (**A**), SDS-PAGE and activity staining analyses of Cel6A-(His)_6_ in *S. lividans*; 1, Supernatant of *S. lividans*/pZRJ362-*cel6A*-(His)_6_; M, Molecular weight marker; (**B**) SDS-PAGE and activity staining analyses of Cel6A-(His)_6_ in *P. pastoris*; 1, Supernatant of *P. pastoris*/pPICZα-*cel6A*-(His)_6_; M, Molecular weight marker.

It was perhaps because of the endogenous protease of *S. lividans* 1326, the expression pattern of Cel6A-(His)_6_ in *S. lividans* 1326 was different from that in *P. pastoris*. As shown in Figure 6A, Some minor proteins (thin arrows), were thought to be derived from Cel6A-(His)_6_ by proteolytic degradation. This result was consistent with previous observations, which also indicated the generation of proteolytic products in the supernatants of *S. lividans* transformants [1,14]. These degradation products of Cel6A-(His)_6_ also exhibited CMCase activity (Figure 6A, the right hand figure), and this could be probably due to the retention of the catalytic domain of Cel6A. In contrast, no proteolytic product was detected in the supernatant of *P. pastoris* carrying pPICZα-*cel6A*-(His)_6_ (Figure 6B). As shown in Figure 6B, there were a wide range of clear bands (thin arrow) which maybe a variety of the glycosylated Cel6A-(His)_6_, and there were also some proteins which may be stained by Congo red (big arrow).

## 3. Experimental Section

### 3.1. Strains, Plasmids and Culture Conditions

For cloning, pMD19-T vector (TaKaRa, Dalian, China) and *E. coli* DH5α (Tiangen, Beijing, China) were used, restriction endonucleases and T4 DNA Ligase were purchased from New England Biolabs (Ipswich, MA, USA). For expression, *P. pastoris* GS115 and pPICZα-C vector were purchased from Invitrogen (San Diego, CA, USA). Genomic DNA from *T. fusca* YX was purchased from ATCC (catalog BAA-629D-5). *S. lividans* 1326 and pZRJ362 constructed in this paper were used for heterologous protein production. Methylation-deficient *E. coli* ET12567/pUZ8002 was used to transfer DNA from *E. coli* into *S. lividans* by conjugation. *S. lividans* 1326 and transformants were grown at 30 °C on mannitol soya flour agar for sporulation. For the production of Cel6A, *S. lividans* transformants carrying expression plasmid were inoculated in tryticase soy broth (TSB) medium (OXOID CM129, Hampshire, UK) with continuous shaking (200 rpm). When applicable, antibiotics were used to select recombinant strains including 100 μg/mL Ampicillin, 50 μg/mL Kanamycin, 25 μg/mL Chloramphenicol and 5 μg/mL Thiostrepton which were purchased from Sigma (St. Louis, MO, USA). For production of Cel6A in *P. pastoris*, yeast extract and peptone were obtained from OXOID (Basingstoke, Hampshire, UK), yeast nitrogen base (YNB) without amino acids was purchased from BD (Sparks, MD, USA), biotin was obtained from Amersco (Solon, OH, USA), and Zeocin was purchased from Invitrogen (San Diego, CA, USA).

### 3.2. Construction of Expression Plasmid pZRJ362

The plasmid pZRJ362 for protein expression using *S. lividans* as a host was constructed as follows. The plasmid pSP72 (Promega, Madison, WI, USA) was digested with *Xba*I and *Eco*RI to obtain the fragment carrying *ori* (*E. coli*) and Ampicillin (Amp) resistance gene *bla*. The plasmid pHZ1358, which was kindly provided by K. Bao and Z. Deng (Shanghai Jiao Tong University, Shanghai, China), was also digested with *Xba*I and *Eco*RI to obtain the fragment containing *ori*T of RP4, *tsr* gene for Thiostrepton resistance and the replication (*rep*) ORF of pIJ101 for autonomous replication of the plasmid in *Streptomyces*. The two fragments were mutually ligated to generate pZRJ. Afterwards, the synthetic fragment of *Xba*I-Xi promoter-Shine-Dalgarno sequence-MCS-*amyA*2 terminator-*Sac*I was subcloned into the *Xba*I-*Sac*I gap of the plasmid pZRJ, yielding the expression vector pZRJ362. The gene fragment of Xi promoter sequence encoded the promoter of xylose isomerase of *Actinoplanes missouriensis* and the *amyA*2 fragment encoded the terminator region of amylase from *Streptomyces avermitilis* MA-4680. Diverse restriction sites were available within the MCS to facilitate gene cloning, including *Nhe*I, *Kpn*I, *Afl*II, *Afe*I, *Mlu*I and *Bam*HI. All the constructs were sequenced by Invitrogen (Shanghai, China).

### 3.3. Cloning of Endoglucanase Gene *cel6A* in *Streptomyces lividans*

The *cel6A* gene including the native signal peptide was PCR amplified from genomic DNA of *T. fusca* YX with the primers *cel6A-f* (5′-GCTAGCTCCCCCAGACCTCTTCGCGCTCTTC-3′) and *cel6A-r* (5′-GGATCCTCAGCTGGCGGCGCAGGTAAGGGTCG-3′), which included *Nhe*I and *Bam*HI restriction sites (underlined), respectively. To simplify the purification process, we introduced (His)_6_-tag into the *C*-terminus of recombinant Cel6A. To obtain His-tagged Cel6A, the reverse primer *cel6A-His* (5′-GGATCCTCAGTGGTGGTGGTGGTGGTGGCTGGCGGCGCAGGTAAGGGTCG-3′) was used. These two PCR products were cloned in plasmid pMD19-T. After the digestion of *Nhe*I and *Bam*HI, 1.6 kb fragments were ligated into the *Nhe*I-*Bam*HI gap of pZRJ362 to generate the expression vectors pZRJ362-*cel6A* and pZRJ362-*cel6A*-(His)_6_, respectively. From the latter expression vector, the corresponding protein was produced with a *C*-terminal 6× His tag.

### 3.4. Cloning of Endoglucanase Gene *cel6A* in *Pichia pastoris*

To achieve the target gene encoding the mature region of endoglucanase Cel6A from *T. fusca* YX genome and obtain His-tagged *cel6A* gene, three primers (*cel6A-F*, *cel6A-R*, *cel6A-His-R*) were designed. The mature *cel6A* gene was amplified by PCR with *cel6A-F* (5′-ATCGATGAATGATTCTCCGTTCTACGTC-3′) as forward primer and *cel6A-R* (5′-TCTAGATTATCAGCTGGCGGCGCAGGTAA-3′) as reverse primer with *Cla*I and *Xba*I restriction sites denoted as the underline, respectively. To obtain His-tagged *cel6A*, the reverse primer *cel6A-His-R* (5′-TCTAGATTATCAATGATGATGATGATGATGGCTGGCGGCGCAGGTAA-3′) was used with *Xba*I restriction site and (His)_6_-tag denoted as the underline too. The PCR products were cloned into pMD19-T, yielding plasmids pMD19-*cel6A* and pMD19-*cel6A*-(His)_6_, respectively.

To achieve secretive expression, the *P. pastoris*-*E. coli* shuttle vector pPICZα-C and recombinant plasmid pMD19-*cel6A*/pMD19-*cel6A*-(His)_6_ were both digested with *Cla*I and *Xba*I and mutually ligated. Afterwards transformants were selected on the low salt LB agar plates (5 g/L yeast extract, 10 g/L tryptone, 5 g/L NaCl, 15 g/L agar, and adjusted pH to 7.5) containing 25 μg/mL Zeocin. Then recombinant plasmids designated as pPICZα-*cel6A* and pPICZα-*cel6A*-(His)_6_ were achieved, respectively. After being digested with *Pme*I, the linearized pPICZα-*cel6A* and pPICZα-*cel6A*-(His)_6_ were transformed into *P. pastoris* GS115 by electroporation using MicroPulser (Bio-Rad, Hercules, CA, USA) under the PIC setting. The *P. pastoris* transformants were selected at 30 °C on the YPDS agar plates (10 g/L yeast extract, 20 g/L peptone, 20 g/L dextrose, 20 g/L agar and 1 M sorbitol) containing 100 μg/mL Zeocin for 3–4 days. Then, the multi-copy transformants were screened by using YPDS agar plates containing higher Zeocin concentration (2000 μg/mL) and incubated at 30 °C for 2 days. Finally, the multi-copy transformants were checked for the integration by genomic PCR.

### 3.5. Production and Purification of Cel6A

For the production of heterologous protein Cel6A or Cel6A-(His)_6_, one spore of each *S. lividans* 1326 carrying the corresponding expression vector was inoculated into a test tube containing 5 mL of TSB medium supplemented with 5 μg/mL Thiostrepton, followed by cultivation at 30 °C for 3 days. Then 1 mL of the preculture medium was seeded into a 250 mL baffled flask containing 100 mL TSB medium with 5 μg/mL Thiostrepton, and incubated at 30 °C for 4–7 days.

For the production of heterologous Cel6A or Cel6A-(His)_6_ from *P. pastoris*, a single colony was inoculated into 25 mL BMGY medium (10 g/L yeast extract, 20 g/L peptone, 100 mM potassium phosphate (pH 6.0), 13.4 g/L YNB, 4 × 10^−4^ g/L biotin, 1% (*v*/*v*) glycerol) in a 250 mL baffled flask and shaken (220 rpm) at 29 °C for 18 h. Then the cells were harvested by centrifuging at 3000× *g* for 5 min and resuspended in 100 mL BMMY medium (10 g/L yeast extract, 20 g/L peptone, 100 mM potassium phosphate (pH 6.0), 13.4 g/L YNB, 4 × 10^−4^ g/L biotin, 0.5% (*v*/*v*) methanol) in a 500 mL baffled flask, shaken (220 rpm) for 4 days. To maintain induction, 100% methanol was added to a final concentration of 0.5% during the induction phase.

To purify the His-tagged interest protein, the mycelium was separated from the supernatant by filtrating with 0.22 μm Millex-GP Filter Unit (Merck Millipore, MA, USA). Afterwards, the His-tagged recombinant protein was purified from the supernatant using a Ni Sepharose™ 6 Fast Flow (GE Healthcare, Little Chalfont, UK) column according to the standard procedures of the manufacturer.

### 3.6. Endoglucanase Activity Assays

The cells were harvested at a selected time point, afterwards the cell extracts were centrifuged and the activities of culture supernatants were measured. The dinitrosalicyclic acid (DNS) method [26] was used to determine endoglucanase activity with low-viscosity carboxymethylcellulose (CMC; Sigma, St. Louis, MO, USA) as cellulose substrate. Subsequently, 450 μL of 1% CMC in acetate buffer (50 mM, pH 6.5) was placed into test tubes and incubated in a waterbath at 55 °C for 5 min, then 50 μL culture supernatants or properly diluted supernatants were added, vortexed and incubated for 30 min, and 750 μL DNS solution (1% 3,5-dinitrosalicyclic acid, 20% potassium sodium tartrate, 1% NaOH, 0.2% phenol, 0.05% Na_2_SO_3_) was added and vortexed. Subsequently, samples were boiled for 10 min, and the absorbance of 200 μL sample at 540 nm was measured on iMark Microplate Reader (Bio-Rad, Hercules, CA, USA). One enzyme unit (U) is defined as an average of 1 μmol of glucose equivalent released per min in the assay reaction. All the enzyme activity values presented were averages obtained from triplicate measurements.

### 3.7. SDS-PAGE and Endoglucanase Activity Staining

SDS-PAGE analyses were performed to detect the extracellular proteins. Activity staining was used for visualization of carboxymethyl-cellulase (CMCase) activity bands on the gel with slightly modifications [27]. The proteins were separated on a 12% separating gel with 0.15% CMC-Na. The gel was washed twice for 30 min each with cold wash solution (acetate buffer (50 mM, pH 6.5) containing 25% isopropanol) to remove the sodium dodecyl sulfate. After that, the gel was washed three times for 30 min each with cold acetate buffer (50 mM, pH 6.5). Then the gel was transferred to acetate buffer (50 mM, pH 6.5) and incubated at 55 °C for 1 h. Finally, the gel was stained in 0.2% Congo Red for 30 min and destained in 1 M NaCl solution. Clear bands against the red background indicated the degradation of CMC-Na.

## 4. Conclusions

In this study, combining the xylose isomerase promoter and amylase terminator, we have constructed a *Streptomyces*-*E. coli* shuttle vector pZRJ362. *T. fusca* YX endoglucanase Cel6A was highly expressed as secretory proteins in *S. lividans* 1326 using this novel expression vector. A most remarkable experimental finding was the higher capacity of *S. lividans*/pZRJ362 to produce *Thermobifida* heterologous protein compared to *P. pastoris*. This newly constructed vector may be applicable to efficient production of other actinomycetes enzymes.
